# Interruption of onchocerciasis transmission in Bioko Island: Accelerating the movement from control to elimination in Equatorial Guinea

**DOI:** 10.1371/journal.pntd.0006471

**Published:** 2018-05-03

**Authors:** Zaida Herrador, Belén Garcia, Policarpo Ncogo, Maria Jesus Perteguer, Jose Miguel Rubio, Eva Rivas, Marta Cimas, Guillermo Ordoñez, Silvia de Pablos, Ana Hernández-González, Rufino Nguema, Laura Moya, María Romay-Barja, Teresa Garate, Kira Barbre, Agustín Benito

**Affiliations:** 1 National Centre for Tropical Medicine, Health Institute Carlos III (ISCIII in Spanish), Madrid, Spain; 2 Network Biomedical Research on Tropical Diseases (RICET in Spanish), Madrid, Spain; 3 Ministry of Health, Malabo, Equatorial Guinea; 4 National Center of Microbiology, Institute of Health Carlos III, Madrid, Spain; 5 Department of Preventive Medicine, University Hospital Nuestra Señora de la Candelaria, Tenerife, Spain; 6 National School of Health, Institute of Health Carlos III, Madrid, Spain; 7 Department of Preventive Medicine, University Hospital of Mostoles, Madrid, Spain; 8 National Program for Onchocerciasis and other Filariasis Control, Ministry of Health, Malabo, Equatorial Guinea; 9 Jimenez Diaz Foundation University Hospital, Madrid, Spain; 10 Neglected Tropical Disease Support Center, Task Force for Global Health, Atlanta, Georgia, United States of America; University of Buea, CAMEROON

## Abstract

**Background:**

Onchocerciasis, also known as river blindness, is a parasitic disease. More than 99 percent of all cases occur in Africa. Bioko Island (Equatorial Guinea) is the only island endemic for onchocerciasis in the world. Since 2005, when vector *Simulium yahense* was eliminated, there have not been any reported cases of infection. This study aimed to demonstrate that updated WHO criteria for stopping mass drug administration (MDA) have been met.

**Methodology/Principal findings:**

A cross-sectional study was conducted from September 2016 to January 2017. Participants were 5- to 9-year-old school children. Onchocerciasis/lymphatic Filariasis (LF, only in endemic districts) rapid diagnostic tests (RDTs) were performed. Blood spots were collected from RDT positive children and 10 percent of the RDT negatives to determine Ov16 and Wb123 IgG4 antibodies through enzyme-linked immunosorbent assay (ELISA). Skin snips were collected from RDT positives. Filarial detection was performed by PCR in positives and indeterminate sera. Black fly collection was carried out in traditional breeding sites. A total of 7,052 children, ranging from 5 to 9 years of age, were included in the study. Four children (0.06%) were Ov16 IgG4 RDT positives, but negative by ELISA Ov16, while 6 RDT negative children tested positive by ELISA. A total of 1,230 children from the Riaba and Baney districts were tested for LF. One child was Wb123 RDT positive (0.08%), but ELISA negative, while 3 RDT negative children were positive by Wb123 ELISA. All positive samples were negative by PCR for onchocerciasis and LF (in blood spot and skin snip). All fly collections and larval prospections in the traditional catching and prospection sites were negative.

**Conclusions/Significance:**

WHO criteria have been met, therefore MDA in Bioko Island can be stopped. Three years of post-treatment surveillance should be implemented to identify any new occurrences of exposure or infection.

## Author summary

Onchocerciasis, commonly called river blindness, is a chronic parasitic disease particularly prevalent in Africa. It is transmitted through the bites of infected *Simulium* blackflies. Onchocerciasis is endemic in Equatorial Guinea. Huge achievements have been made in human and vector control during the last two decades, especially on Bioko Island. Eliminating onchocerciasis transmission on Bioko is feasible given its isolation from other landmasses, which also reduces the risk of reinvasion by the disease vector. Recently updated WHO guidelines for stopping mass drug administration (MDA) and verifying elimination of human onchocerciasis (2016) established a new critical threshold to verify elimination of onchocerciasis transmission based on novel serological tests. We applied these techniques in a representative sample of 5- to 9-year-old school children. An entomological assessment was also carried out. We found no evidence of current infection or recent transmission. There was no evidence of onchocerciasis vectors, and our results from the sample population meet the current WHO serologic criteria for stopping MDA. Based on these results, we recommended to the Ministry of Health and Social Welfare of Equatorial Guinea that MDA on Bioko Island be stopped and that 3 years of post-treatment surveillance should be undertaken to identify any new occurrences of exposure or infection.

## Introduction

Onchocerciasis is a parasitic disease caused by the filarial worm *Onchocerca volvulus*. It is transmitted through the bites of infected *Simulium* blackflies, which breed in fast-flowing streams and rivers. Symptoms include rashes, severe itching and various skin lesions, and blindness. The disease is endemic in 31 countries in sub-Saharan Africa, two countries in Latin America, and in Yemen. An estimated 18 million people are infected with the disease and have dermal microfilariae. 99% of the infected individuals live in Africa [1,2].

Human onchocerciasis is one of the two filarial helminth “neglected tropical diseases” targeted for geographically local elimination [3]. In the Americas, onchocerciasis elimination has traditionally been considered feasible as most onchocerciasis foci were confined and usually small. Since 2013, the World Health Organization (WHO) has certified four countries in Latin America as free of human onchocerciasis [4]. In Africa, where onchocerciasis has been endemic over vast areas, with highly efficient vectors and much higher endemicity levels, elimination was not initially considered to be feasible [5].

The Onchocerciasis Control Programme in West Africa (OCP) was launched in 1974 by the World Health Organization (WHO), followed by the African Programme for Onchocerciasis Control (APOC), initiated in 1995. Both programs established mass treatment with ivermectin combined with aerial spraying of breeding sites with selected insecticides in fast-flowing rivers as principal methods for controlling onchocerciasis [3]. Great progress has been made towards elimination. In most OCP/APOC countries, nationwide onchocerciasis elimination now seems to be an obtainable objective [6]. This paradigm shift from control to elimination occurred in 2012 due to success in Latin America, the shift to integrated NTD control, and the successful elimination of onchocerciasis in some parts of Africa [7].

The island of Bioko is part of the Republic of Equatorial Guinea and is the only island in the world where onchocerciasis is endemic [8]. Initially, the island was considered free of loiasis. The presence of intermediate hosts [9], and the recent reporting of an imported case of loiasis in a US traveler returning from the island [10] call for attention and further research. Two out of four districts have reported cases of Lymphatic Filariasis (LF). In 1990, several control activities were launched by the OCP, including long-term ivermectin mass treatment in all 52 island communities [11,12]. Afterwards, APOC became the sponsoring agency and introduced community-directed treatment with ivermectin (CDTI) throughout the island in 2000 [8]. In addition, a vector elimination project started when APOC was established in 1995. A feasibility study was carried out in 1996, confirming the high vectorial efficiency of the endemic Bioko form of S*imulium yahense* [13] and the distribution of the vector breeding sites [14]. From 2001 to 2005, a large-scale larviciding trial using ground-based applications was undertaken using helicopter and ground-based applications of temephos [8,15]. In 2005, the endemic Bioko form of *S*. *yahense* was finally eliminated from the island. Since then, there has not been any reported transmission or any serious epidemiological situation in Malabo City or elsewhere on the island [8]. According to personal communication from the Ministry of Health and Social Welfare of Equatorial Guinea (MINSABS in Spanish), the last MDA with ivermectin was administered in 2012 in urban Malabo and in 2016 elsewhere on the island.

A 2014 cross sectional study found no positive MF skin snip assessment in 544 study participants ages 5 years and older [16,17]. In the recently updated WHO guidelines for stopping mass drug administration and verifying elimination of human onchocerciasis (2016), new tools were proposed to verify the transmission interruption, stop CDTI and begin post-treatment surveillance. Following the WHO updated recommendations, the objectives of this study were: a) to verify onchocerciasis transmission interruption in Bioko Island, Equatorial Guinea; b) to validate a methodology to assess Ov16 prevalence in children younger than 10 years of age and; c) to develop a protocol to verify onchocerciasis elimination that can be applied in other African countries with hypoendemic intensity of transmission or where mass drug administration (MDA) has been conducted for a number of years. Data on LF were also collected as part of the study.

## Methods

### Study area

The Island of Bioko is part of the Republic of Equatorial Guinea, which also includes Rio Muni on the mainland and the island of Annobon. It is located in the Bay of Guinea in Central Africa, about 40 km southwest of the Cameroon coast. Bioko Island covers an area of approximately 2,017 km2 (779 square miles). It is 72 km (44.7 miles) long and is divided into four districts (Malabo, Luba, Riaba and Baney). Bioko Island has 334,463 inhabitants. Most of the population is concentrated on the northern part of the island in the Malabo district, where Malabo, the capital of Equatorial Guinea is located [18]. Tropical rainforest covers much of the interior of the island and the topography is characterized by steeply sloping volcanoes and calderas. Bioko Island has a humid tropical climate with an average annual temperature of 25°C and two distinct seasons: a dry season from November to March and a rainy season from April to October.

### Study design and participants

A cross-sectional study was conducted from September 2016 to January 2017. The eligible participants were 5- to 9-year old school children who had lived in Bioko Island for the past three years. According to the most recent WHO guidelines, a sample size of 1,100 to 2,000 children younger than 10 years of age per administrative unit is required for Ov-16 serology testing in order to detect a prevalence of less than 0.1% at the upper bound of the 95 percent confidence interval. When the eligible population of children is less than 1,100, all eligible children are to be tested [19].

First, we obtained an updated census report on school-aged children from the Ministry of Education. According to this data, sampling was not required in two districts (Riaba and Luba), since the population was below 1,100. All the children from the Baney district and rural Malabo who met the inclusion criteria were included in the study because the numbers were lower than first estimated. In urban Malabo, a random sampling method was implemented.

A second visit took place in May 2017 to obtain a second RDT and blood spot for ELISA/PCR in patients whose results were RDT/ELISA positives and/or were in the detection threshold limit.

Entomological surveys were carried out in the rivers with potential breeding sites.

### Procedures

Two teams, consisting of three local technicians, one supervisor and two coordinators were assembled and trained before the field work began.

Prior to starting the study, a comprehensive field training program was provided along with training on the proper use of diagnostic tools. The participation questionnaire was pre-tested one week before the beginning of the project. Questionnaires were provided to each school administrator, who distributed them to the children before the science team came to the school to conduct the testing. Children were instructed to take the forms home and have their parents or guardians complete them before they were returned. At least two reminders were given before the testing date. The form required the participant’s sex, birthdate, age, and asked whether the child had lived in Bioko Island for the past three years and if the child had ever take ivermectin. Test results were later recorded in the same document.

The geographic coordinates of each school (latitude and longitude) were collected using the offline maps application MAPS.ME.

This study combined the use of serological tests based on recombinant antigens with the later confirmation of diagnostic results by molecular diagnosis. The SD BIOLINE Onchocerciasis IgG4 rapid diagnostic test, manufactured and distributed by Standard Diagnostics, Inc. (SD), was performed following the product instructions. Further information on this technique has been described elsewhere [20]. Sterile techniques were used to obtain finger-prick blood samples. Fingers were cleaned using an alcohol swab and sterile cotton balls. Technicians wore disposable gloves. All materials were safely disposed of. In those districts considered co-endemic to lymphatic Filariasis and onchocerciasis (Riaba and Baney), SD BIOLINE LF IgG4 RDT was also performed, together with SD BIOLINE onchocerciasis/LF IgG4 biplex.

Blood spots were collected from all the children with either a positive Ov RDT or a positive LF RDT and from a random sample of 10 percent of the negative RDT children for determination of Ov16 and Wb123 IgG4 antibodies through enzyme-linked immunosorbent assay (ELISA). This subsample (10% of negative RDT) was randomly selected by equal probability systematic sampling. Every tenth RDT negative child was selected for further blood spots collection.

Sterile techniques were used to place finger-prick blood spots onto circles on 5 x 5 cm Whatman 903 protein saver cards. Skin samples were obtained near the iliac crest of each individual through Walser matrix forceps. Samples, stored and transported at 4°C, were delivered to the National Center of Microbiology (NCM) laboratory at the Institute of Health Carlos III in Madrid for further analysis.

An ELISA protocol was run to detect anti-Ov16 IgG4 antibodies in the eluted blood from the Whatman filter paper (S1 ELISA Protocol). Plates were sensitized with 0.5 μg/ml of Ov16 recombinant protein, obtained and purified as described in Hernández-Gónzalez et al., 2016 [16]. A poly-His tail was added to the carboxy-terminus of the Ov-16 sequence amplified from a vector (kindly donated by Professor JE Bradley, School of Life Sciences and University of Nottingham, UK). Then, the construction was subcloned into pGEX-6P-1 plasmid (GE Healthcare, Little Chalfont, UK). Further expression and purification steps were undertaken as explained in the indicated paper. Two positive controls were included: (i) an anti-Ov16 human recombinant monoclonal antibody (hIgG4, Bio-Rad) and (ii) a pool of positive sera from patients with onchocerciasis (clinical and parasitological confirmation). A standard curve from each positive control was used to identify positive samples on each plate allowing comparisons among plates and days. The anti-Ov16 IgG4 mAb positive control was diluted as follows: 12, 6, 3, 2, 1.5 and 1 ng/ml and in the case of the positive sera, pool dilutions ranked from 1/400 to 1/3.200. The cut- off for the recombinant positive control (anti-OV16 mAb; stock: 2.9 mg / ml), was set at 2.01 ng / ml following the indications of Golden et al., 2017 [21]. The dilution 1/800 from the pool of positive sera was chosen as the cut-off, with optical densities similar to those obtained with the anti-Ov16 mAb at 2.01 ng/ml.

A similar ELISA protocol was run to detect anti-Wb123 IgG4 antibodies. The only difference in methodology was that wells were sensitized with Wb123 recombinant protein. The Wb123- pUC57 construction, corresponding to the GenBank HQ438580 sequence, was obtained from Genscript (Piscataway, NJ, USA) and further directionally subcloned in pQE-30 expression vector. Isopropyl β-D-1-thiogalactopyranoside (IPTG) 0.01 mM was used to induce protein expression with ON incubation at 16°C. The Wb123 recombinant protein was purified by Ni2+- Sepharose 4B affinity chromatography (GE Healthcare) in native conditions and according to the manufacturer’s recommendations. Finally, the Wb123 recombinant antigen was dialyzed against PBS and quantified using the Pierce BCA Protein Assay Kit (Thermo Scientific Rockford, IL, USA). A standard curve was developed; in this case, with serial dilutions (1/400 to 1/3,200) of a *Wuchereria bancrofti* positive sera pool (WHO collection provided by Dr. N. Weiss, Swiss Tropical Institute, Basel). The chosen cut-off was the OD corresponding to the 1/1600 control sera dilution.

In both ELISAs (Ov16 and Wb123), the sera were classified as positive, indeterminate or negative, following the criteria described below:

- Indeterminate sera: those with an Optical density (O.D.) ± 10 percent of cut-off value (gray area).
- Positive sera: those with an O.D. above the upper limit of the gray area.
- Negative sera: those with an O.D. below the lower limit of the gray area.

Molecular protocols (PCR) were undertaken to confirm serological positive samples: blood spots for FL and onchocerciasis positives and skin snip for *Onchocerca* positives. DNA extraction from skin snips specimens and the blood spots were performed with the QIAamp DNA mini kit (QIAGEN, IZASA, Madrid). Filarial detection was carried out by two independent Polymerase chain reaction (PCR) methods. A real-time PCR, modified by Tang et al. (2010) [22], targets the internal transcribed spacer in one region of the ribosomal gene (ITS-1), which enables the identification of positive samples by post reaction analysis by melting temperature (Tm) curve of the amplified fragments (Tm = 77.50°C ± 1.0°C) and by the size of the amplified fragment (344bp for *O*. *volvulus*, 312bp for *Mansonella spp*., 301bp for *W*. *bancrofti* and 286bp for *Loa loa*) and a conventional PCR targeting of the mitochondrial COI gene [23] (see further details in S1 Table). All samples were made in duplicate.

PCR products were analyzed by automatic electrophoresis (QIAxcel, QIAGEN, IZASA, Madrid) or conventional electrophoresis in 2 percent agarose gels stained with Pronosafe (Pronadisa, Madrid). The confirmation of the filarial species was performed by sequencing the amplified fragment using the Big Dye Terminator v3.1 Cycle Sequencing Kit (Applied Biosystems, Massachusetts, EEUU) on an ABI PRISM 3700 DNA analyzer (Applied Biosystems, Massachusetts, USA). Previously, PCR products were purified using the Illustra DNA and Gel Band Purification Kit (General Electric Healthcare, Little Chalfont, UK). All amplified products were sequenced twice in both directions.

All sites accomplishing APOC experts’ criteria for harboring larvae of the simulids were visited. Black fly collection was carried out in representative catching sites by one team in each community, consisting of two fly collectors and a human attractant (human bait). The human bait method involved having the individual sit in one place barefoot for up to one hour. During this worktime, the fly collector exposed his legs and caught all landing *Simulium* flies with the help of an exhaustor (“pooter”). Collections began, local time, at 0700 and ended at 1700. Collectors received ivermectin 1 week before beginning the collection process.

### Statistical analysis

Individual data, RDT and laboratory results were analyzed to obtain frequencies of each variable. Prevalence (with 95 percent confidence intervals) of onchocerciasis and LF were calculated from RDT results. In three out of the four districts (and rural Malabo), all the eligible children attending school the day of the visit were included. It was not confirmed whether any children were absent on the day of the survey or whether any local children were not attending school at all. Prevalence and confidence limits were computed following the recommendations of Brown et al. for interval estimation for a binomial proportion [24]. The Wilson interval for small n (when n<100), and the interval suggested in Agresti and Coull for larger n (when n≥100), were used.

A univariate analysis was performed to explore if any variable showed a significant relationship with positive onchocerciasis RDT cases. Analyses were performed using the statistical package Stata 14.0. Schools were mapped using the free software QGIS version 2.18.7.

### Ethics statement

The study was approved by the MINSABS on Bioko Island and the research ethics and animal welfare committee at the Health Institute Carlos III (ISCIII in Spanish) in Spain, under the number CEI PI 22_2016-v3. The school headmasters and children´s parents/guardians were informed of the day of the visit and the scope of the study by an official letter from the MINSABS. Written informed consent was obtained from all parents or guardians prior to study inclusion. Data were analyzed anonymously.

## Results

**Fig 1:**
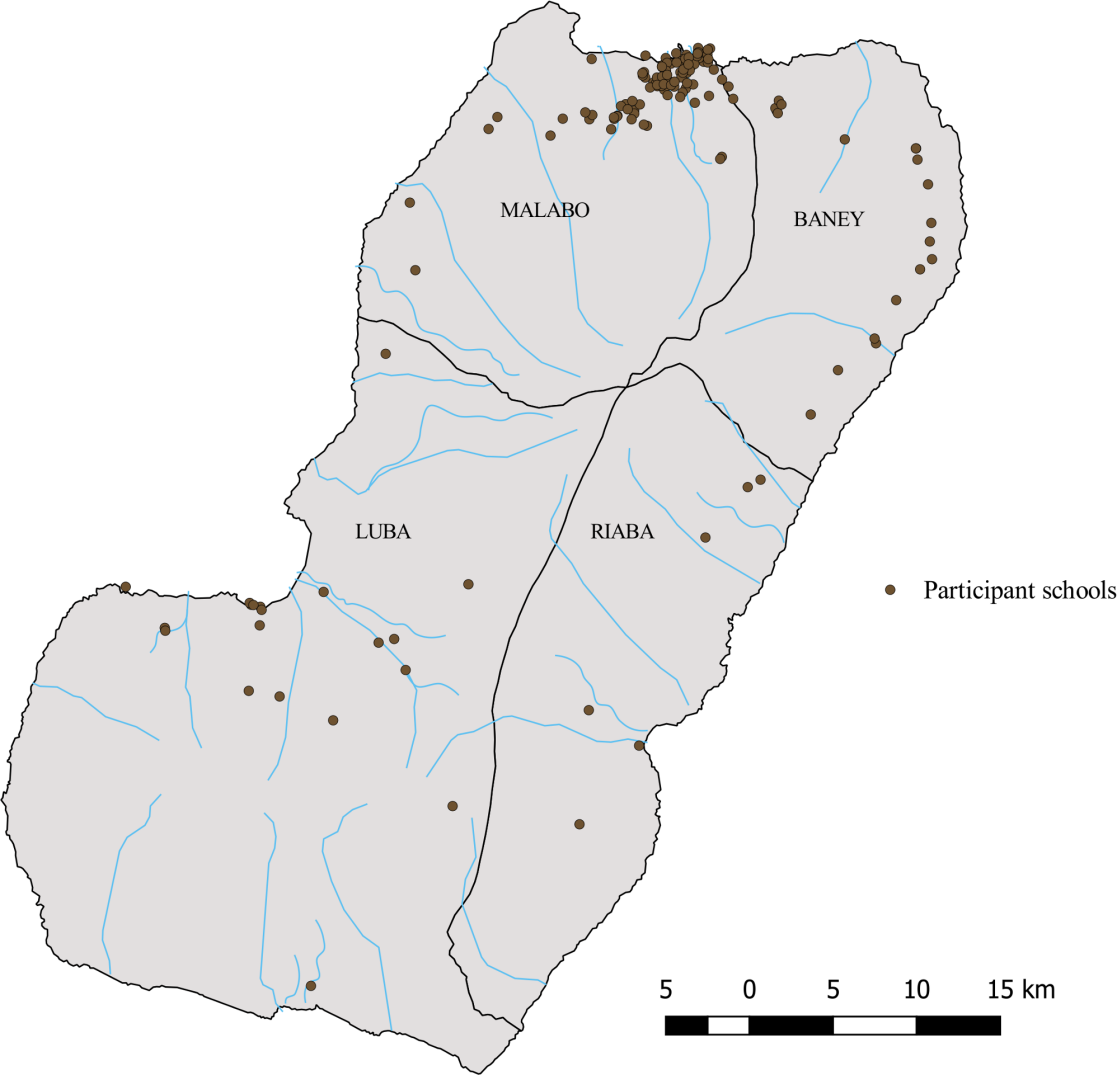
Geographical distribution of participant schools in Bioko Island, September 2016 to January 2017.

A total of 7,052 school children, ages 5 to 9 years old and living in Bioko Island for the last three years or more, participated in the epidemiological assessment. The study sample was recruited from 147 schools, distributed all around the Island (Fig 1). In the Riaba, Luba and Baney districts and rural Malabo, all of the eligible 5- to 9-year-old children agreed to participate and were thus included. In urban Malabo, several parents did not sign the study agreement form (around 25%), and replacements for these individuals were found to obtain a sample of 4,342 randomly selected children.

The majority of the 5- to 9-year-old children lived in urban Malabo. Overall, ages were evenly distributed. The sample was 52.4% female. 96 percent of the respondents reported that their children had never received ivermectin (Table 1).

**Table 1:** Characteristics of the sample by district, Bioko Island, September 2016 to January 2017.

Variable		Total (n = 7,052)		Riaba (n = 95)		Baney (n = 1,154)		Luba (n = 493)		Rural Malabo (n = 968)		Urban Malabo (n = 4,342)	
		n	%	n	%	n	%	n	%	n	%	n	%
**Sex**	**Male**	3,357	47.60	44	46.3	590	51.1	254	51.5	453	46.8	2,016	46.4
	**Female**	3,695	52.40	51	53.7	564	48.9	239	48.5	515	53.2	2,326	53.6
**Age**	**5**	1,305	18.51	16	16.8	160	13.9	75	15.2	205	21.2	849	19.6
	**6**	1,479	20.97	28	29.5	256	22.2	87	17.6	230	23.8	878	20.2
	**7**	1,431	20.29	21	22.1	236	20.5	96	19.5	199	20.6	879	20.2
	**8**	1,519	21.54	15	15.8	264	22.9	128	26.0	165	17.0	947	21.8
	**9**	1,318	18.69	15	15.8	238	20.6	107	21.7	169	17.5	789	18.2
**History of ivermectin treatment**	**Yes**	283	4.01	1	1.1	74	6.4	15	3.0	47	4.9	146	3.4
	**No**	4,601	65.25	78	82.1	575	49.8	450	91.3	541	55.9	2,957	68.1
	**Don´t know**	2,168	30.74	16	16.8	505	43.8	28	5.7	380	39.3	1,239	28.5
**Skin snip specimen collected**	**Yes**	4	0.06	1	1.1	0	0.0	0	0.0	3	0.3	0	0.0
	**No**	7,048	99.94	94	98.9	1,154	100.0	493	100.0	965	99.7	4,342	100.0
**Whatman paper sample collected**	**Yes, positive RDT**	5	0.07	1	1.1	1	0.1	0	0.0	3	0.3	0	0.0
	**Yes, 10% random of negative RDT**	715	10.14	8	8.4	116	10.1	47	9.5	97	10.0	443	10.2
	**No**	6,332	89.79	86	90.5	1,037	89.9	446	90.5	868	89.7	3,899	89.8

### Onchocerciasis

**Fig 2:**
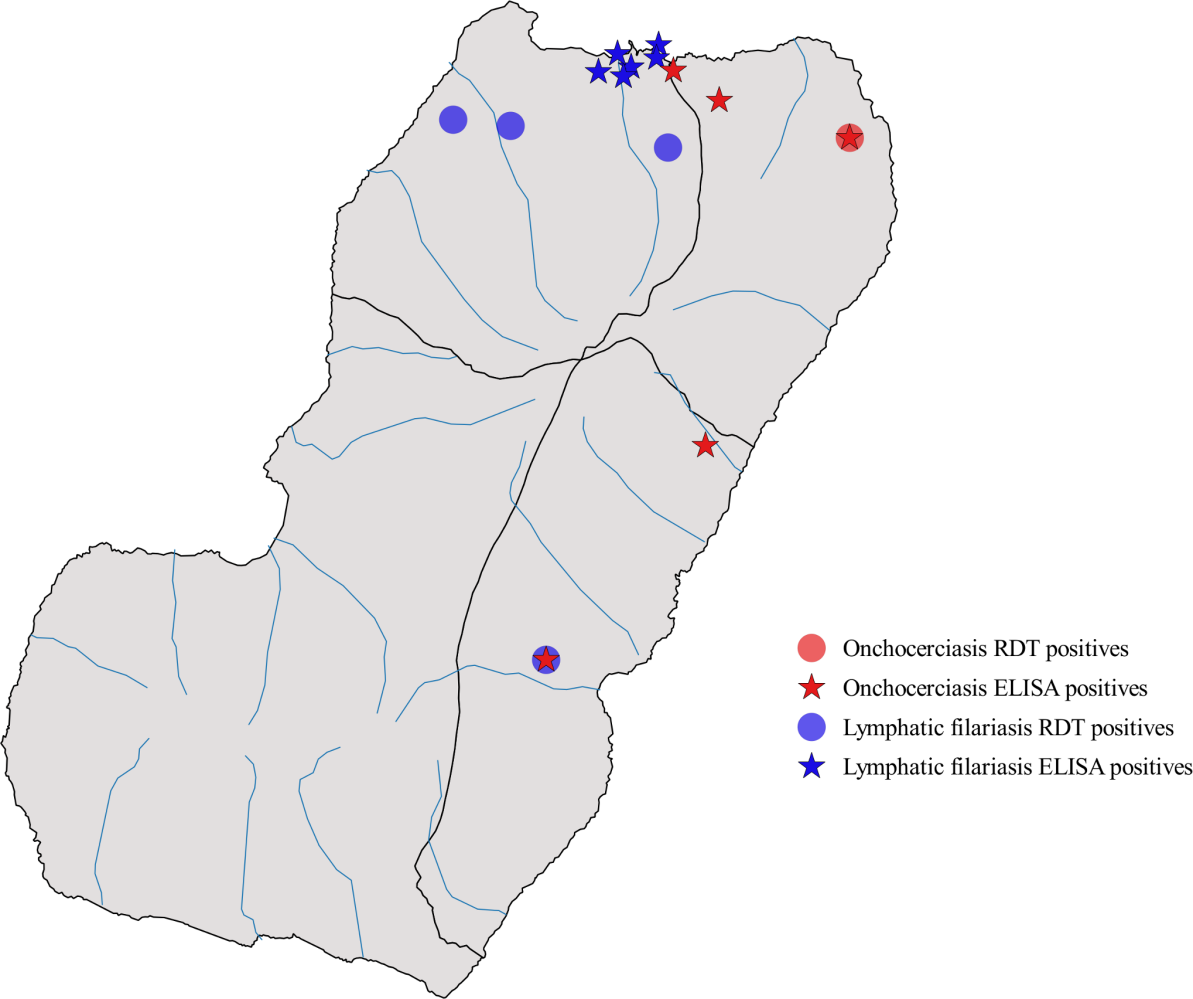
Geographical distribution of RDT and ELISA positives in 5- to 9-year-old school children in Bioko Island, September 2016 to January 2017.

Finger-prick blood samples for Ov16 IgG4 RDT were obtained from all 7,052 school children. From the overall sample, four children (0.06 percent) were found to be positive for Ov16 IgG4 antibodies by RDT (95 percent CI upper limit = 0.11). One was from the Riaba district and three lived in rural Malabo (Fig 2). No significant association with onchocerciasis RDT positive cases was found for any variable, except for district. Rural Malabo presented the highest frequency of onchocerciasis RDT positive cases (n = 3; p<0.001).

Blood spots were collected from 720 school children. Five of them were obtained from positive individuals and 715 (10 percent) were collected from a randomized selection of RDT negative children. Skin snips were obtained from the RDT positive children (n = 4). The 4 onchocerciasis RDT positive children were negative by ELISA Ov16, while 6 RDT negative children from urban Malabo tested positive to onchocerciasis by ELISA (prevalence = 0.83 percent; 95 percent CI = 0.34–1.85). Three samples were considered as indeterminate sera, because they were close to the ELISA cutoff threshold. PCR analysis was negative for all positive/indeterminate blood and skin samples (n = 13; Table 2).

**Table 2:** Prevalence of onchocerciasis in children less than 10 years old tested by Ov-16 IgG4 RDT, ELISA and PCR, Bioko Island, September 2016 to January 2017.

Population	Onchocerciasis RDT (n = 7,052)				Onchocerciasis ELISA Ov16 (n = 720)				Onchocerciasis PCR (n = 720)			
	n	Positive	Prev. (%)	95% CI	n	Positive	Prev. (%)	95% CI	n	Positive	Prev. (%)	95% CI
**Overall population**	**7,052**	**4**	**0.06**	**(0.02–0.14)**	**720**	6*	**0.83**	**(0.34–1.85)**	**720**	**0**	**0**	**(0.00–0.64)**
**Riaba**	95	1	1.05	(0.18–5.72)	11	0	0	(0.00–25.88)	11	0	0	(0.00–25.88)
**Baney**	1,154	0	0	(0.00–0.40)	120	0	0	(0.00–3.73)	120	0	0	(0.00–3.73)
**Luba**	493	0	0	(0.00–0.93)	49	0	0	(0.00–7.27)	49	0	0	(0.00–7.27)
**Rural Malabo**	968	3	0.31	(0.06–0.95)	96	0	0	(0.00–3.85)	96	0	0	(0.00–3.85)
**Urban Malabo**	4,342	0	0	(0.00–0.11)	444	6*	1.33	(0.55–2.99)	444	0	0	(0.00–1.03)

* 3 indeterminate sera were not included as positive; Prev: prevalence; 95% CI: 95% confidence interval; N.P.: not performed.

### Lymphatic filariasis

A total of 1,230 children from Riaba and Baney were tested for lymphatic Filariasis. In one Baney district school (with n = 19), the LF IgG4 RDT was not performed due to logistical problems. One 9-year-old girl (0.08%) from Baney was found positive for Wb123 by LF IgG4 RDT (Table 3), while no child tested positive for Wb123, according to onchocerciasis/LF IgG4 biplex results.

**Table 3:** Prevalence of lymphatic filariasis in children younger than 10 years old tested by LF IgG4/biplex RDT, ELISA and PCR, Bioko Island, September 2016 to January 2017.

Population	Lymphatic Filariasis RDT*(n = 1,230)				Lymphatic Filariasis Wb123 ELISA (n = 131)				Lymphatic Filariasis PCR** (n = 6)			
	n	Positive	Prev. (%)	95% CI	n	Positive	Prev. (%)	95% CI	n	Positive	Prev. (%)	95% CI
**Overall population**	1,230	1	0.08	(0.00–0.51)	131	5	3.82	(1.64–8.62)	131	0	0	(0.00–3.42)
**Riaba**	95	0	0	(0.00–3.89)	11	2	18.18	(5.14–47.70)	11	0	0	(0.00–25.88)
**Baney**	1,135	1	0.09	(0.00–0.55)	120	3	2.50	(0.53–7.41)	120	0	0	(0.00–3.73)

* By LF IgG4 RDT and/or onchocerciasis/LF IgG4 biplex.

**PCR was performed in RDT positive (n = 1) and ELISA positives (n = 5).

Samples were processed by ELISA with recombinant Wb123. The LF RDT positive child was ELISA negative, while 3 children from Baney and 2 from Riaba who tested negative by RDT tested positive when sera was processed by Wb123 ELISA (Fig 2). One onchocerciasis RDT positive child was also positive to LF by Wb123 ELISA. All LF serologically positive children (n = 6) were negative by PCR (Table 3).

### Verification of results

In May 2007, a second serum sample was obtained from 16 children who were either RDT positive (Onchocerciasis or LF) or ELISA positive/indeterminate for confirmatory test (n = 18). Two school children were unavailable during the second visit.

Out of the above population, 2 children were onchocerciasis RDT positive (prevalence = 0.03%; 95% CI = 0.00–0.11), 1 by Ov16 IgG4 RDT and the other by Oncho/LF biplex IgG4 RDT. Both were negative by Ov16 ELISA and blood spot/skin snip PCR. The child, who was previously found positive to LF, was negative by biplex RDT in this second round. The second Ov16-IgG4 ELISA analysis was negative for all the samples (n = 16) except one. Regarding those samples that were positive by ELISA with recombinant Wb123 in the preliminary analysis (n = 5), 3 remained positive, 1 became negative and the fifth one had been collected from one of the two unavailable children. Skin snips were obtained from the confirmed biplex RDT (n = 2) and Ov16 ELISA (n = 1) positive children. PCR analysis was negative for all of them (Table 4; S2 Table).

**Table 4:** Prevalence of onchocerciasis and lymphatic Filariasis in children less than 10 years old tested by RDT, ELISA and PCR, on the second visit, Bioko Island, May 2017.

Diagnostic test	Initial sample	Positive samples obtained during the second visit (n = 16)	Overall prevalence (%)	95% CI*
**Onchocerciasis RDT**	**7,052**	2	0.03	(0.00–0.11)
**Onchocerciasis ELISA Ov16 (10%negative + RDT positive)**	**720**	1	0.14	(0.00–0.86)
**Onchocerciasis PCR (serologically positive)**	**13**	0	0	(0.00–22.81)-
**Lymphatic Filariasis RDT**	**1230**	0	0	(0.00–0.38)-
**Lymphatic Filariasis Wb123 ELISA (10%negative + RDT positive)**	**131**	3**	2.29	(0.48–6.81)
**Lymphatic Filariasis PCR (serologically positive)**	**6**	0	0	(0.00–39.03)-

* For the 95% CI estimation, an unknown population was included as denominators

** One previously Wb123 ELISA positive child was unavailable during the second visit.

Updated WHO guidelines established that serologically positive children found negative by PCR testing of skin snips are considered negative for patent infection with *O*. *volvulus* and are accepted as not contributory to the 0.1% threshold calculation [19]. According to this criterion, no child was positive for onchocerciasis (Table 2). The 2 RDT and the remaining ELISA positive child (according to the sera obtained on the second visit) were considered as *O*. *volvulus* “exposed.” The MINSABS will re-examine them 1 to 1.5 years after the first visit to determine if they have developed patent infection. If so, they will be treated accordingly, following WHO recommendations [19].

### Entomological assessment

**Fig 3:**
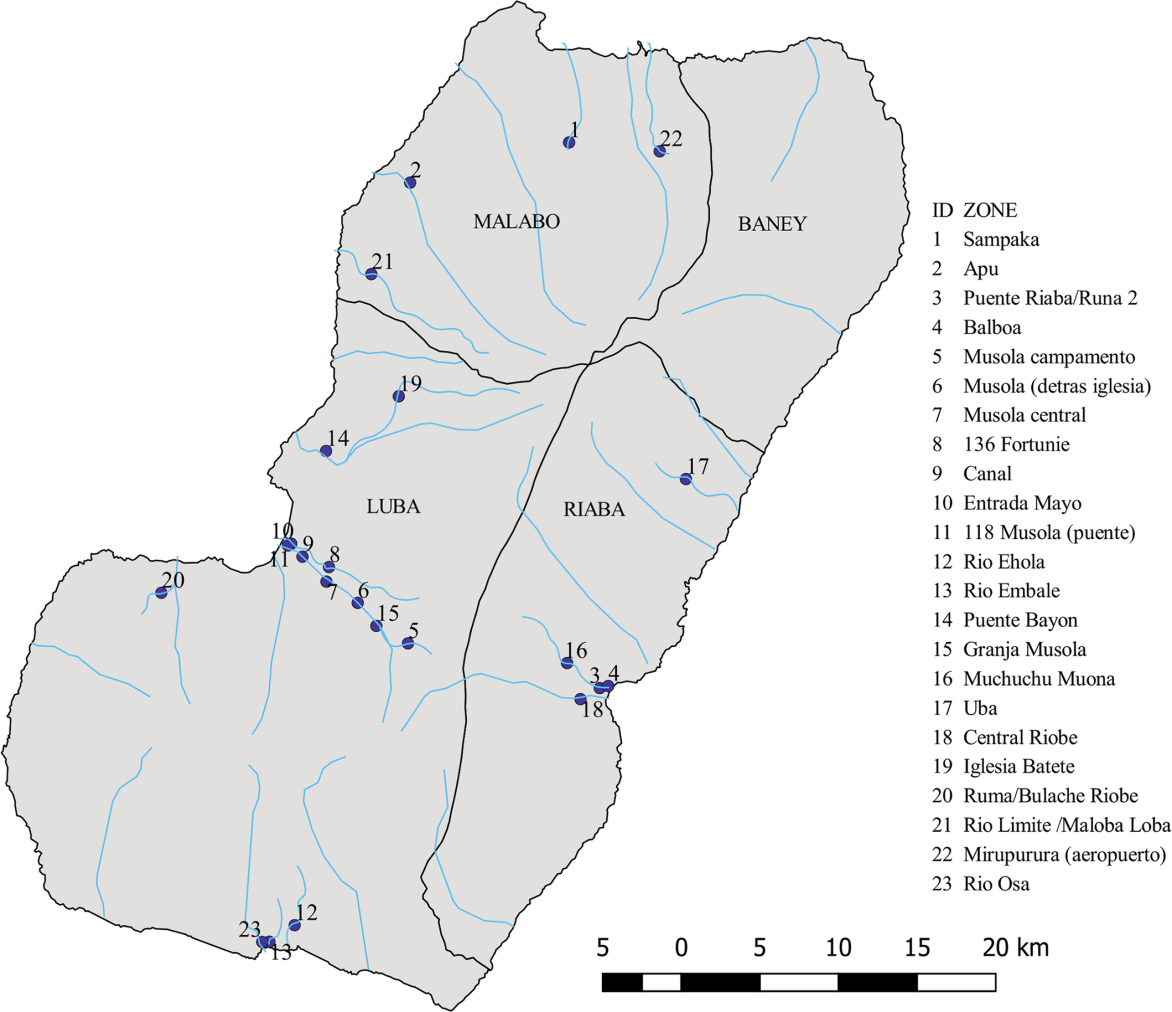
Entomological survey of potential breeding sites, Bioko Island, September 2016 to February 2017.

Entomological assessment was performed from the 29th of August to the 23rd of September, 2016. Due to fieldwork difficulties and the need for extra personnel, the Ureka area (breeding sites placed in rivers Mohaba, Ehola and Osha) was visited in February 2017 (Fig 3). All fly collection and breeding site prospection were negative.

## Discussion

We conducted an evaluation of *O*. *volvulus* transmission in Bioko Island, Equatorial Guinea, guided by updated WHO guidelines [19]. We found no evidence of current infection or recent transmission: there was no evidence of onchocerciasis vectors, and our results in 5- to 9-year-old children meet the current WHO 0.1 percent serologic criteria for stopping MDA. Moreover, none of the children tested positive when RDT was combined with ELISA. Based on these results, we recommended to the Ministry of Health and Social Welfare in Equatorial Guinea (MINSABS) that MDA in Bioko Island be stopped and that 3 years of post-treatment surveillance should begin, to identify any new occurrences of exposure or infection.WHO defines disease elimination as the reduction to zero of the incidence of infection caused by a specific pathogen in a defined geographical area, with minimal risk of reintroduction, as a result of deliberate efforts [25]. To measure the progress towards elimination, three phases have been defined for onchocerciasis control programs. The phase 1 or intervention phase is characterized by regular ivermectin treatment with a minimum requirement of 80 percent therapeutic coverage. This phase typically lasts at least 12 to 15 years, which corresponds to the reproductive lifespan of the adult worm when exposed to drug pressure [19]. Phase 2, also called “post-treatment surveillance” follows the intervention phase and typically lasts 3 to 5 years. The final phase (phase 3) starts at the end of the 3 to 5 years of post-treatment surveillance and is also known as “post-elimination surveillance.” It follows the confirmation of the initial assessments at the end of phase 2, thereby providing strong evidence that transmission has been permanently eliminated in a country. According to our results, Bioko Island is currently in phase 2. On Bioko Island, initial onchocerciasis control activities began in 1990, using ivermectin distributed by mobile teams [12,26]. After 8 years, a significant reduction in onchocerciasis prevalence (from 74.5 to 38.4 percent), and community MF load (from 28.3 to 2.3) [11] was achieved. Afterwards, APOC became the sponsoring agency and introduced CDTI throughout the island in 2000 [8]. Since then, therapeutic coverage has ranged from 51 [11] to 75 percent [27]. Although the therapeutic coverages have been relatively low, treatment intervention has been steady for more than 20 years. On the other hand, Equatorial Guinea (Bioko island in particular) is one of the three African countries (Uganda and the United Republic of Tanzania being the other two) that supplemented MDA together with vector control [19]. Furthermore, given that Bioko Island has been free of onchocerciasis vectors for the past 12 years [3,8] and the insular isolation of Bioko (32 km from the Cameroon coast and 400 km from the Guinean mainland coast), WHO might reconsider if the post treatment surveillance is truly needed in this case.

This study also reports the first use of anti-Ov16 IgG4 RDT in a primary field screening followed by confirmatory ELISA/PCR in the laboratory to certify onchocerciasis elimination in an African endemic area. Several markers for infection have been used for measuring onchocerciasis disease burden; however, most of them seem to be insensitive to certifying elimination [28]. Commonly used methods for diagnosing *O*. *volvulus* infections (microscopic detection of microfilariae (MF) in skin snips and nodule palpation) are insensitive, especially when MF skin densities are low. PCR of the skin snips may provide greater sensitivity but still require sampling skin snips. Moreover, the cost of performing PCR on entire populations, or even in sentinel groups, is high [29]. An alternative approach is applying antibody detection to *O*. *volvulus* specific antigens that are expressed by the larval stages of the parasite. This method seems to be particularly useful to identify incident infections in communities having already undergone MDA [30]. Several serological markers for exposure to onchocerciasis have been assessed in the past [31,32]. The most widely used and the one adopted as a tool for monitoring onchocerciasis control and elimination in the Americas is the Ov-16 antigen [33]. This test has been mainly performed by enzyme immunoassay (EIA or ELISA), that detects IgG4 to this antigen. IgG4 detection results are more reliable than IgG detection, leading to fewer false positive results, which is critical for its use in a low prevalence, elimination scenario [30]. In Sub-Saharan Africa, the Ov16 ELISA was used to prove that onchocerciasis transmission was interrupted in the Wadelai focus of Northwestern Uganda [34] and the Abu Hamed focus, in Sudan [35].

In this new paradigmatic scenario, where elimination seems obtainable, a standardized affordable, simple and rapid alternative for testing in the field is necessary. To fill this gap, a new RDT was recently developed by PATH [20,29]. To date, this Ov16 RDT accuracy has been mainly assessed in surveillance studies and/or African focus with high onchocerciasis prevalence. Its usefulness in defining when to stop MDA is still limited [29,36,37]. Our study is the first one to provide evidence on its accuracy and usefulness in an African focus with low-prevalence conditions prevailing at the end-stage of control programs.

Combining this method with other diagnostic strategies (ELISA in 10% of RDT negatives and RDT positives, and PCR in all seropositives), we also contribute to a better understanding of the dynamics of Ov16 antibody responses for accurate interpretation of seroprevalence data. In our study, four children (0.06%) were positive by RDT, but negative by ELISA and PCR. This prevalence was even lower after verification of results during a second visit (0.03 percent). Regarding the Onchocerciasis and Lymphatic Filariasis IgG4 biplex RDT, it also performed well in the present study. One positive child out of 1,230 (0.08 percent) was deemed negative by ELISA and PCR. The integration of two filarial antigens in one device is of added value in co-endemic areas where MDA has been ongoing for several years. It saves time, money, and human resources in regions where co-endemicity is suspected. Nevertheless, it may not provide accurate results in areas that are highly endemic for loiasis [38], which was not the case on Bioko Island. Therefore, the high sensitivity and specificity shown by both RDTs support their usefulness to determine the interruption of onchocerciasis transmission.

In summary, we have developed and tested a protocol for stopping MDA and evaluating onchocerciasis elimination following the updated WHO guidelines [19]. Its implementation on Bioko Island has been a success, but some considerations should be taken into account before applying it to other African countries. Previous research has proven that onchocerciasis elimination with ivermectin treatment might be feasible in some endemic foci in Africa [39,40]. In Bioko island, MDA was quite successful, but interruption of the disease transmission might have been partially or completely due to the disappearance of the *Simulium* vectors, as was the case in Uganda [34]. In the case of this study, the elimination of the vector, combined with the isolated nature of Bioko and the long history of MDA, were responsible for a particularly low baseline level. This should be taken into account before using this protocol in other contexts.

### Limitations and conclusions

Our study has several limitations. First, the lower participation rate in urban Malabo may affect the interpretation of the results in this particular district. Moreover, the school attendance rates for 5- to 9-year-old children were not provided by the Ministry of Education (probably this information is not systematically recorded). Nevertheless, the sample size was larger than would have been required in this administrative unit according to the WHO guidelines. Second, the recombinant positive control antibody AbD19432_hIgG4 was complex to standardize, due to the reagent instability when it was diluted to the final concentration to be used in ELISAs. Secondary extractions from indeterminate serological samples (within the detection limit threshold) were obtained in May 2017 to verify the results. Only one of the conflictive samples requested yielded a positive absorbance value by both positive controls (recombinant control and serum control). Third, pre-analytical bias due to the complex field logistics might exist. To reduce these potential biases, detailed guideline and standard operational procedures (SOPs) were prepared and piloted prior to the field work. All the SOPs were also tested by internal control before and during the field work.

In conclusion, WHO criteria have been met in Bioko Island. Consequently, MDA can be stopped and 3 years of post-treatment surveillance should begin to identify any new occurrences of exposure or infection. Successful elimination of onchocerciasis infection throughout Equatorial Guinea may be a feasible goal for the relatively near future. To that end, a survey extending to the continental area is needed before talks about countrywide elimination begin.

## Supporting information

S1 Checklist STROBE checklist. (DOC)

S1 ELISA Protocol Ov16 / Wb123 Protocol. (DOCX)

S1 Table PCR procedures, Bioko Island, Equatorial Guinea. (DOCX)

S2 Table Summary of positive and indeterminate results in the first (September-December 2016) and second visits (May 2017), Bioko Island, Equatorial Guinea. (DOCX)
