# Supplementary material for: Interruption of onchocerciasis transmission in Bioko Island: Accelerating the movement from control to elimination in Equatorial Guinea
Source: PLoS Negl Trop Dis. 2018 May 3;12(5):e0006471. doi: 10.1371/journal.pntd.0006471 (PMC5953477; doi:10.1371/journal.pntd.0006471)
Supplement: S1 ELISA Protocol — (DOCX) [file pntd.0006471.s002.docx]

**ELISA Ov16 / Wb123 PROTOCOL**

1. **Blood spots elution.** Eight 3 mm diameter disks of saturated blood samples on Whatman filter paper were punched and placed in 200 µl of dilution buffer (Phosphate Buffered Saline (PBS) - 0.05% Tween 20 - 3% skim milk) and left at 4ºC overnight.
2. **Coating.** 96-well polystyrene plates (Nunc MediSorp , Denmark) were sensitized with the purified recombinant protein (0.5μg/ml; 100μl/well) in a carbonate buffer with a pH 9.6 and left overnight at 4°C. Ov16 o Wb123 recombinant protein was used as the antigen source for each independent ELISA.
3. **Washing**. Plates were washed three times with 300 µl PBS pH 7.4 0.05% Tween 20 (PBS-T).
4. **Blocking.** 200 µl of dilution buffer were added per well and incubated for 1 hour at 37°C.
5. **Washing.** Plates were washed as indicated in step 3.
6. **Samples and controls.**
7. The eluted blood samples were diluted at ½ in dilution buffer and added to the corresponding wells in duplicate (100 µl/well).
8. The clone AbD19432_hIgG4 anti-Ov16 recombinant monoclonal antibody (human IgG4) supplied by Bio-Rad was used as a positive control at the following concentrations: 12 ng/ml, 6 ng/ml, 3 ng/ml, 2 ng/ml, 1.5 ng/ml and 1 ng/ml in dilution buffer and added to the correspondent wells in duplicate (100 µl/well).
9. A pool of positive sera from patients with onchocerciasis was included as a positive control. Dilutions from 1/400 to 1/3200 were prepared in the dilution buffer and added to the corresponding wells in duplicate (100 µl/well).

The samples and controls were incubated for 1 hour at 37°C.

1. **Washing.** Plates were washed five times with 300 µl PBS pH 7.4 0.05% Tween 20 (PBS-T).
2. **Secondary antibody**. A mouse anti-human IgG4 labeled with horseradish peroxidase (9200-05, Southern Biotech) was used at 1/4000 in a dilution buffer (100μl/well). The plates were incubated for 1 hour at 37°C.
3. **Washing.** Plates were washed as described in Step 3.
4. **Developing and reading**. After the addition of 100μl/well of SureBlue TMB Microwell reagent Peroxidase Substrate (52-00-00, KPL) and incubation on a plate shaker for 6 minutes, the reaction was halted with 100μl of 0.5 M H_2_SO_4_. The plates were read at 450 nm using a 620 nm filter.

Standard curves from each positive control were used to identify positive samples on each plate, allowing comparisons between plates and days.
