# Supplementary material for: Interruption of onchocerciasis transmission in Bioko Island: Accelerating the movement from control to elimination in Equatorial Guinea
Source: PLoS Negl Trop Dis. 2018 May 3;12(5):e0006471. doi: 10.1371/journal.pntd.0006471 (PMC5953477; doi:10.1371/journal.pntd.0006471)
Supplement: S1 Table — (DOCX) [file pntd.0006471.s003.docx]

| PCR name | Tª alig. | Nº cycles | Primer Name | Seq 5'→3' | Final conc. |
| --- | --- | --- | --- | --- | --- |
| RT-PCR Fil-ITS | 50ºC | 45 | FIL2-F | GGTGAACCTGCGGAAGGATC | 0.2 μM |
|  |  |  | FIL 2-Loa | GGTGAACCTGCRGMWGGATC | 0.2 μM |
|  |  |  | FIL2-R | TGCTTATTAAGTCTACTTAA | 0.375 μM |
| PCR-Fil-COI | 50ºC | 40 | NTF | TGATTGGTGGTTTTGGTAA | 0.2 μM |
|  |  |  | NTR | ATAAGTACGAGTATCAATATC | 0.2 μM |
| Tª. Alig.: Alignment temperature of primers on PCR; Final conc. : Final concentration of primers use don PCR. | | | | | |
