# Supplementary material for: Interruption of onchocerciasis transmission in Bioko Island: Accelerating the movement from control to elimination in Equatorial Guinea
Source: PLoS Negl Trop Dis. 2018 May 3;12(5):e0006471. doi: 10.1371/journal.pntd.0006471 (PMC5953477; doi:10.1371/journal.pntd.0006471)
Supplement: S2 Table — (DOCX) [file pntd.0006471.s004.docx]

| **ID** | **DISTRICT** | **10% RANDOM SAMPLE** | **FIELD WORK (September-December 2016)** | | | | | **SECOND VISIT (May 2017)** | | | | |
| --- | --- | --- | --- | --- | --- | --- | --- | --- | --- | --- | --- | --- |
|  |  |  | **monoplex RDT** | **biplex RDT** | **ELISA OV16** | **ELISA Wb123** | **PCR** | **monoplex RDT** | **biplex RDT** | **ELISA OV16** | **ELISA Wb123** | **PCR** |
| **1** | RURAL MALABO | NO | POS. | N.P. | NEG. | NEG. | NEG. | POS. | N.P. | NEG. | N.P. | NEG. |
| **2** | RURAL MALABO | NO | POS. | N.P. | NEG. | NEG. | NEG. | NEG. | N.P. | NEG. | N.P. | NEG. |
| **3** | RURAL MALABO | NO | POS. | N.P. | NEG. | NEG. | NEG. | NEG. | N.P. | NEG. | N.P. | NEG. |
| **4** | URBAN MALABO | YES | NEG. | N.P. | POS. | NEG. | NEG. | NEG. | N.P. | NEG. | N.P. | NEG. |
| **5** | URBAN MALABO | YES | NEG. | N.P. | POS. | NEG. | NEG. | NEG. | N.P. | POS. | N.P. | NEG. |
| **6** | URBAN MALABO | YES | NEG. | N.P. | POS. | NEG. | NEG. | NEG. | N.P. | NEG. | N.P. | NEG. |
| **7** | URBAN MALABO | YES | NEG. | N.P. | POS. | NEG. | NEG. | NEG. | N.P. | NEG. | N.P. | NEG. |
| **8** | URBAN MALABO | YES | NEG. | N.P. | POS. | NEG. | NEG. | NEG. | N.P. | NEG. | N.P. | NEG. |
| **9** | URBAN MALABO | YES | NEG. | N.P. | IND. | NEG. | NEG. | NEG. | N.P. | NEG. | N.P. | NEG. |
| **10** | URBAN MALABO | YES | NEG. | N.P. | IND. | NEG. | NEG. | NEG. | N.P. | NEG. | N.P. | NEG. |
| **11** | URBAN MALABO | YES | NEG. | N.P. | IND. | NEG. | NEG. | NEG. | N.P. | NEG. | N.P. | NEG. |
| **12** | BANEY | YES | N.P. | NEG. | NEG. | POS. | NEG. | N.P. | NEG. | NEG. | IND. | NEG. |
| **13** | BANEY | YES | N.P. | NEG. | NEG. | POS. | NEG. | N.P. | NEG. | NEG. | POS. | NEG. |
| **14** | BANEY | YES | N.P. | NEG. | NEG. | POS. | NEG. | N.P. | NEG. | NEG. | POS. | NEG. |
| **15** | BANEY | NO | N.P. | POS. LF | NEG. | NEG. | NEG. | N.P. | NEG. | NEG. | NEG. | NEG. |
| **16** | RIABA | NO | N.P. | POS. OC | NEG. | POS. | NEG. | N.P. | POS. OC | NEG. | POS. | NEG. |
| POS: positive; NEG: negative; IND: indeterminate; OC: onchocerciasis; LF: lymphatic filariasis; N.P.:not performed | | | | | | | | | | | | |
